# Cost-effectiveness in an interprofessional training ward within a university department for internal medicine: a monocentric open-label controlled study of the A-STAR Regensburg

**DOI:** 10.3389/fpubh.2024.1340953

**Published:** 2024-08-09

**Authors:** Sophie Schlosser-Hupf, Elisabeth Aichner, Marcus Meier, Sheila Albaladejo-Fuertes, Anna Mahnke, Kirstin Ruttmann, Sophia Rusch, Bernhard Michels, Alexander Mehrl, Claudia Kunst, Stephan Schmid, Martina Müller

**Affiliations:** ^1^Department of Internal Medicine I, Gastroenterology, Hepatology, Endocrinology, Rheumatology and Infectious Diseases, University Hospital Regensburg, Regensburg, Germany; ^2^Nursing Center 4, University Hospital Regensburg, Regensburg, Germany; ^3^Nursing Development Department of the Care Management Head Office, University Hospital Regensburg, Regensburg, Germany

**Keywords:** interprofessional, training ward, cost-effectiveness, economic outcome, cost analysis, interprofessional education, interprofessional collaborative practice, internal medicine

## Abstract

**Introduction:**

Interprofessional collaboration in healthcare involves diverse professionals working together to address complex patient needs. Interprofessional training wards offer workplace-based interprofessional education in real healthcare settings, fostering collaborative learning among students. While their educational value is widely recognized, debates persist regarding their cost-effectiveness due to limited research. This study assesses the cost efficiency of the interprofessional training ward Regensburg (A-STAR) within the Department of Internal Medicine I at the University Hospital Regensburg, compared to conventional wards.

**Methods:**

From October 2019 to December 2022, 7,244 patient cases were assigned to A-STAR or conventional wards by case managers, with a comprehensive analysis of all associated revenues and costs.

**Results:**

A-STAR treated 1,482 patients, whereas conventional wards treated 5,752 patients, with more males and younger patients at A-STAR. A-STAR achieved higher profit per case (€1,508.74) attributed to increased revenues and reduced material costs. It generated an average of €1,366.54 more Diagnosis Related Groups (DRG) revenue per case annually than conventional wards, due to greater medical complexity reflected in a higher case-mix index (CMI: 2.4 vs. 2.2). The increased case complexity led to longer patient stays (9.0 vs. 8.1 days) and fewer cases treated annually at A-STAR (27.4 cases/year vs. 37.8 cases/year). The higher CMI did not result in a higher proportion of patients requiring isolation. A-STAR exhibited a higher capacity utilization rate (87.1% vs. 83.9%). Personnel costs per case at A-STAR were initially elevated due to enhanced observation by the senior physician but were gradually mitigated by expanding A-STAR’s bed capacity. Material costs were consistently lower on a per-case basis at A-STAR (€1512.02 vs. €1577.12), particularly in terms of medication expenses, indicating more resource-efficient operations. From the A-STAR graduates, 18 individuals were recruited for permanent positions as doctors or nurses over 2 years.

**Conclusion:**

A-STAR demonstrates economic efficiency and stability even during the COVID-19 pandemic. The substantial personnel acquisition is likely influenced by high levels of satisfaction with education and work and is economically relevant in medical staff shortages. These findings provide a compelling rationale for the broader implementation of interprofessional training wards, establishing them as vital platforms for nurturing future professionals.

## 1 Introduction

This study is grounded in the theoretical framework of interprofessional education, which emphasizes collaborative learning and practice among healthcare professionals to improve patient outcomes and healthcare efficiency. Interprofessional collaboration refers to a concerted and coordinated approach to healthcare delivery involving healthcare professionals from different disciplines working together to address patients’ growing complex health needs (1, 2). This approach recognizes that no single healthcare professional can provide all the necessary care for a patient and that collaboration and communication among healthcare professionals are essential to optimize patient outcomes. Interprofessional healthcare aims to improve the quality of care, enhance patient safety, reduce healthcare costs, and improve patient satisfaction (3–15). Interprofessional collaboration also enhances shared decision-making with patients, ensuring their preferences and values are considered, which is crucial for effective and cost-efficient healthcare delivery (16). Most barriers and facilitators identified were at the inter-individual and organizational levels. The main obstacles included a shortage of time and training opportunities, unclear roles and responsibilities, concerns around professional identity, and inadequate communication practices (17).

Interprofessional training wards are specialized facilities within hospitals or medical centers where healthcare students and professionals from disciplines such as medicine, nursing, pharmacy, physical therapy, and social work come together to learn and practice interprofessional collaboration skills (18, 19). These wards offer real-life healthcare settings, where students work together as a team largely independent from but under the supervision of their trainers to provide care to patients (10, 20). This includes conducting patient assessments, developing treatment plans, implementing interventions, and evaluating patient outcomes. They are an ideal instrument for interprofessional teaching because they provide a controlled real-life environment for healthcare professionals from different disciplines to work together as a team and learn from and about each other. Interprofessional training wards promote a better understanding of the professional roles and responsibilities which may result in a more effective and efficient coopration (21–24). Interprofessional training wards typically involve a range of learning opportunities, including simulations, case studies, and debriefing sessions. They are facilitated by experienced educators and clinicians who help students and professionals to develop their interprofessional competencies, professional skills and provide feedback on their performance.

In 2016 the founding members of the Society for Cost and Value in Health Professions Education conceived the *Prato Statement, which* proposes “that the goal of economic analyses in professional and interprofessional education is to create an evidence base toward education that delivers maximum value for a given spend—and that drives education that is sustainable, accessible, and able to meet future healthcare requirements” (25). While there is no doubt that these training wards provide valuable learning experiences, the question of whether they are cost-effective remains. Few studies have examined the costs and benefits of interprofessional teaching, and even fewer interprofessional training wards in the medical context (26–29). There is one notable cost–benefit analysis of a Danish interprofessional orthopedic training ward. In 2009, Hansen et al. published data from the first Danish undergraduate interprofessional training ward at Regional Hospital Holstebro (30). The study compared costs, complications, and quality of life for 134 patients who underwent primary hip or knee replacement surgery on the interprofessional training ward versus a conventional ward. The results showed that the interprofessional training ward was more cost-effective than the conventional one for primary hip and knee replacement surgeries. Moreover, there was no difference in complications or patient-reported quality of life. In 2022, a study by one of the first German interprofessional training ward, HIPSTA, at Heidelberg University Hospital was published, which examined the clinical outcome of the ward’s surgical patient collective (31). Compared to the 465 patients in the conventional wards, the 243 patients in the HIPSTA showed significantly shorter lengths of stay and fewer reoperations, with no difference in terms of postoperative complications, and in-hospital mortality.

Our study represents the first-ever analysis of an interprofessional training ward within the field of internal medicine, specifically focusing on a primarily gastroenterological patient population with complex medical needs. We investigated the hypothesis that the A-STAR operates with the same cost efficiency as the conventional wards of the Department of Internal Medicine I, Gastroenterology, Hepatology, Endocrinology, Rheumatology, and Infectious Diseases, at University Hospital Regensburg.

## 2 Materials and methods

### 2.1 Patients

All patients who had been admitted to the A-STAR and conventional wards at the Department of Internal Medicine I, Gastroenterology, Hepatology, Endocrinology, Rheumatology, and Infectious Diseases, at the University Hospital Regensburg between October 1, 2019, and December 31, 2022, in this period were eligible for inclusion. Note that part of the conventional ward as well as the A-STAR were closed for Christmas holidays between 23rd December and 1st of January of each year. To mitigate potential selection bias, cases admitted and discharged during this holiday period were deliberately excluded between the 20th of December and the 6th of January annually.

### 2.2 Trial design

This study follows a monocentric, open-label, controlled design. No formal randomization procedure occurred, but case managers who were not otherwise involved in the study randomly allocated patients to either the A-STAR or conventional wards, depending on bed availability. Due to the high capacity utilization and frequent isolation requirements for patients with multi-resistant germs with the hepatology focus of the department, no consideration could be given to case severity or interprofessional educational value when allocating patients to the wards.

The trial protocol was approved by an independent ethics committee in Germany (Ethics Committee of the University of Regensburg: 20-1805_1–101). The trial was conducted by the latest version of the Declaration of Helsinki, with the Good Clinical Practice guidelines of the International Conference on Harmonization and relevant German laws and directives.

### 2.3 Treatment

A team of medical professionals and nurses was responsible for care within the conventional wards. Complementing this team, students in their final years and trainees in nursing actively participated in the daily ward operations of the ward. Patients of the A-STAR received care from a team of up to eight medical students in their final years and up to two nursing trainees per shift in their 2nd and 3rd years of training. They were supervised by experienced medical professionals and nurses. Unlike conventional wards, the A-STAR senior physician is present on the ward most of the day and is credited with 1 Full Time Equivalent (FTE). In addition to their work on the conventional wards, the three senior physicians on the conventional wards are also assigned to the outpatient clinic and the intensive care unit. And are credited with 3 FTEs. Selection for the A-STAR team was conducted via letter of motivation and a comprehensive CV by the head of the department and the head of the nursing team. Notably, medical students devoted 8–16 weeks of their last year to the ward, while nursing trainees allocated approximately 4 weeks to the A-STAR.

The A-STAR bed area is seamlessly integrated within the conventional wards. Medical students and nursing trainees collaborate from a shared base, while doctors and nurses in the conventional wards maintain their distinct bases. Throughout the study period, the A-STAR unit encompassed a range of 8–12 beds, while the conventional wards accommodated between 45 and 49 beds. These wards provide care for patients diagnosed with diverse conditions, including gastroenterological, hepatological, infectious, endocrine, and rheumatological diseases (Table 1).

**Table 1:** Structure.

Characteristic	A-STAR	Conventional wards
Beds		
Mean no. 2019	8	49
Mean no. 2020	10	47
Mean no. 2021	11	46
Mean no. 2022	12	45
Senior physicians		
No.	1	3
Full-time equivalent	1	1,5
Residents (mean no.)	1.5	7.1
Mean no. 2019	1	7
Mean no. 2020	1.9	7.2
Mean no. 2021	1.6	7.3
Mean no. 2022	1	6.8
Medical students		
No.	04-Aug	03-Sep
Nurses		
Mean no.	2.2	12.5
Nursing trainees		
No.	4	02-Jun

**Figure 1:**
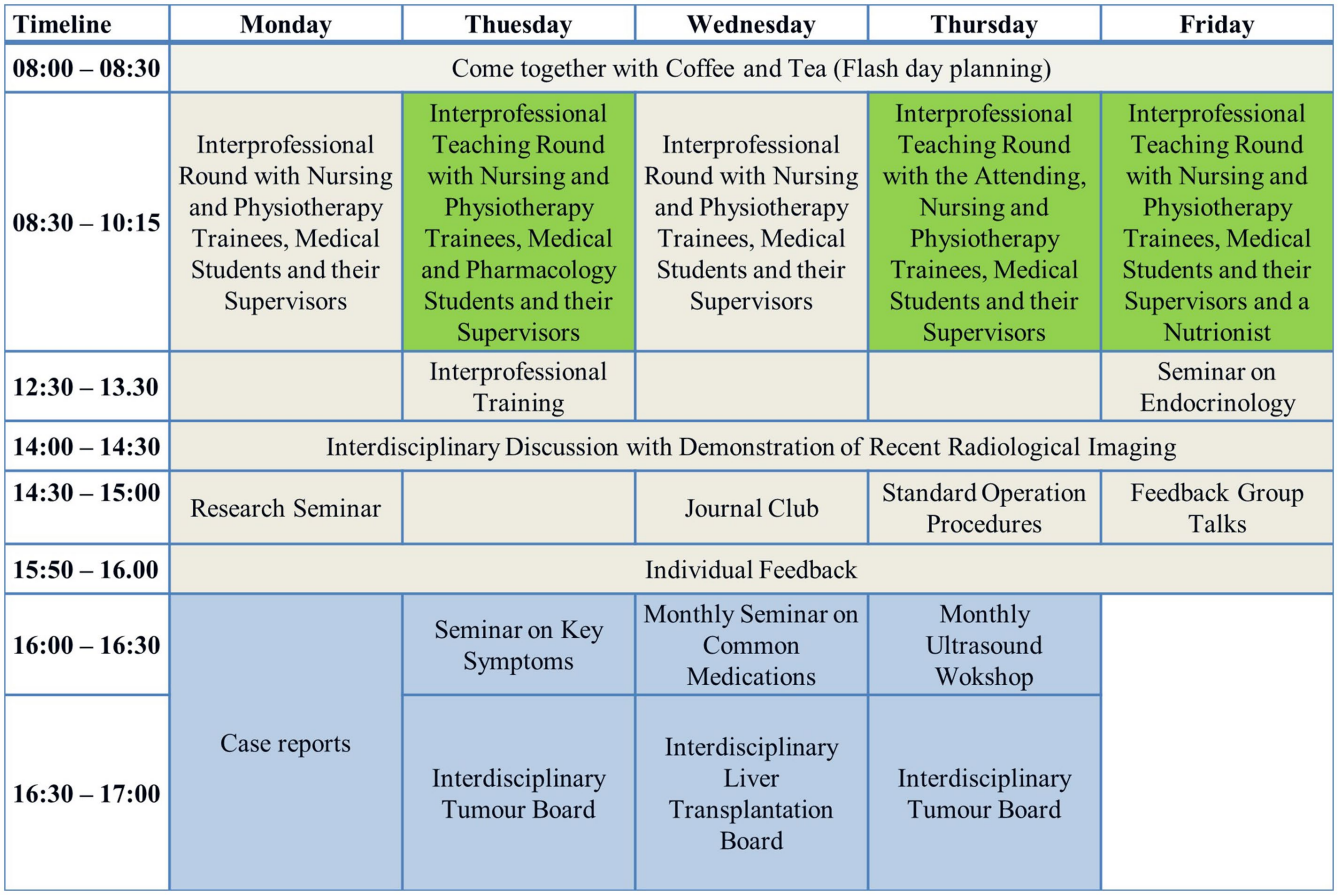
A-STAR schedule. A structured routine characterized the A-STAR activities, encompassing daily planning sessions, patient visits, educational sessions, and feedback discussions.

A structured routine characterized the A-STAR activities, encompassing daily planning sessions, patient visits, educational sessions, and feedback discussions, as illustrated in Figure 1; each day commenced with a unified daily plan after the nursing handover from the night shift and the initial mono-professional tasks performed by nursing trainees. Medical students and nursing trainees conducted the rounds together. On the conventional wards, doctors and nurses aimed to perform the rounds together when possible. Consultations with patients during rounds were primarily conducted by the physicians. Pharmacology students, pharmacists, and nutritionists participated weekly in the A-STAR rounds, evaluating medication for interactions and proper dosages. The conventional wards received advice from colleagues in the pharmacy once a week for selected cases. Weekly teaching visits were facilitated by a medical director or senior medical representative in all wards.

Daily, the A-STAR’s medical students and nursing trainees engage in interprofessional educational training sessions, joined by a diverse spectrum of medical care professionals. Furthermore, the A-STAR provides a comprehensive training repertoire, including specialized offerings such as resuscitation training, practical skills training using models, and in-depth sonography courses, thus ensuring a well-rounded educational experience for its trainees. This collective includes pharmacists, physiotherapists, nutritionists, clinic chaplains, technicians, psychologists, and more. Once a day, the entire medical department team convenes for an interdisciplinary discussion with an interdisciplinary X-ray presentation. Notably, both medical students and A-STAR nursing trainees actively participate in this forum. The day’s activities culminate with feedback discussions and reflections.

### 2.4 End points and assessments

The German Diagnosis-Related Groups (DRG) system is a reimbursement system used in Germany to classify and reimburse hospitals for patient care based on the diagnosis and treatment provided. The system was introduced in 2004 and covers almost all inpatient cases. Under the DRG system, hospitals are paid a lump sum for each case, which is calculated based on the average resource use of selected hospitals. Each DRG is associated with a specific weight that represents the expected resource consumption and cost for treating patients in that group. The case-mix-index (CMI) is a numerical value that reflects the overall mix of patients treated by a hospital during a specific time period, such as a year. It is calculated by summing the individual weights of all patients treated in the hospital and dividing by the total number of patients.

The primary endpoint assessed profit per case, while the secondary endpoints encompassed DRG revenues per case, personnel costs per case, material costs per case, number of cases per bed, bed occupancy rates, and the average length of stay.

Our analysis was conducted on a per-case not per-bed basis to avoid bias by the following facts: 1. Private patients often used double rooms individual instead of shared occupancy. 2. During the COVID-19 pandemic, an area within the wards was temporarily reserved for COVID-19 patients. 3. Due to a shortage of nursing staff, some beds in the A-STAR and conventional wards were temporarily blocked.

Data were drawn from the hospital patient register regarding gender, age, Barthel Index, DRG revenues, number of cases, bed occupancy, and average length of stay. Surcharges, discounts, and revenues for elective medical services were not included in our analysis. The length of stay was calculated for the complete period with a stay in the department’s intensive care unit, if necessary. Medical personnel costs were collected from the current collective agreement. Nursing personnel was not included in the analysis due to the shared nursing pool utilized by both A-STAR and conventional wards. Material costs were requested from the Accounting and Controlling department. Since the material costs of the A-STAR were recorded in total, not per case basis, within the account of one of the conventional wards, these costs were allocated based on the number of beds.

### 2.5 Statistical analysis

Qualitative variables were compared between the A-STAR and the conventional wards by using the chi-square or Fisher’s exact test, as quantitative variables were compared between the A-STAR and the conventional wards by using the Mann–Whitney U test. The Mann–Whitney U test was chosen over the independent t-test due to the non-normal distribution of the data, which was assessed using the Shapiro–Wilk test. All the tests were two-sided, and a *p* value of less than 0.05 was considered to indicate statistical significance. Data analysis was performed with IBM Corp. Released in 2021. IBM SPSS Statistics for Windows, version 28.0. Armonk, NY: IBM Corp.

## 3 Original results

### 3.1 The interprofessional training ward A-STAR: treating more men and younger patients

From October 2019 through December 2022, a total of 7,244 patient cases at the Department of Internal Medicine I, Gastroenterology, Hepatology, Endocrinology, Rheumatology, and Infectious Diseases, at the University Hospital Regensburg were randomly allocated to either the A-STAR or conventional wards by case managers. The demographic characteristics of the patient cases slightly differed in the two treatment groups (Table 2), except for the Barthel Index (*U* = 4191840.000; *Z* = 1.678, *p* = 0.093). Males were significantly more common in the A-STAR group than in the conventional wards [73.5% vs. 70.4%; χ^2^(1) = 5.124; *p* = 0.025]. Patients were significantly younger at the A-STAR (59 yr. vs. 61 yr.; *U* = 4577905.000; *Z* = 3.987; *p* < 0.001).

**Table 2:** Characteristics (2019–2022).

Characteristic	A-STAR (*n* = 1,482)	Conventional wards (*n* = 5,752)
Age		
Median (range)—yr	59 (18–101)	61 (16–98)
Sex		
Male—no. (%)	1,089 (73.5)	4,052 (70.4)
Female—no. (%)	393 (26.5)	1700 (29.5)
Barthel Index		
Median (range)—yr	100 (0–100)	100 (0–100)
Mean number of beds/year	11	46
Mean number of cases/year	300	1738
Mean number of cases/bed/year	27.4	37.8
Mean case mix index/year	2.4	2.2
Mean total DRG revenues	€2,115,448.05	€11,446,987.38
Mean DRG revenues/case	€9,372.83	€8,006.29

### 3.2 The interprofessional training ward A-STAR: superior to conventional wards in annual DRG income and resource-efficient material cost management

**Figure 2:**
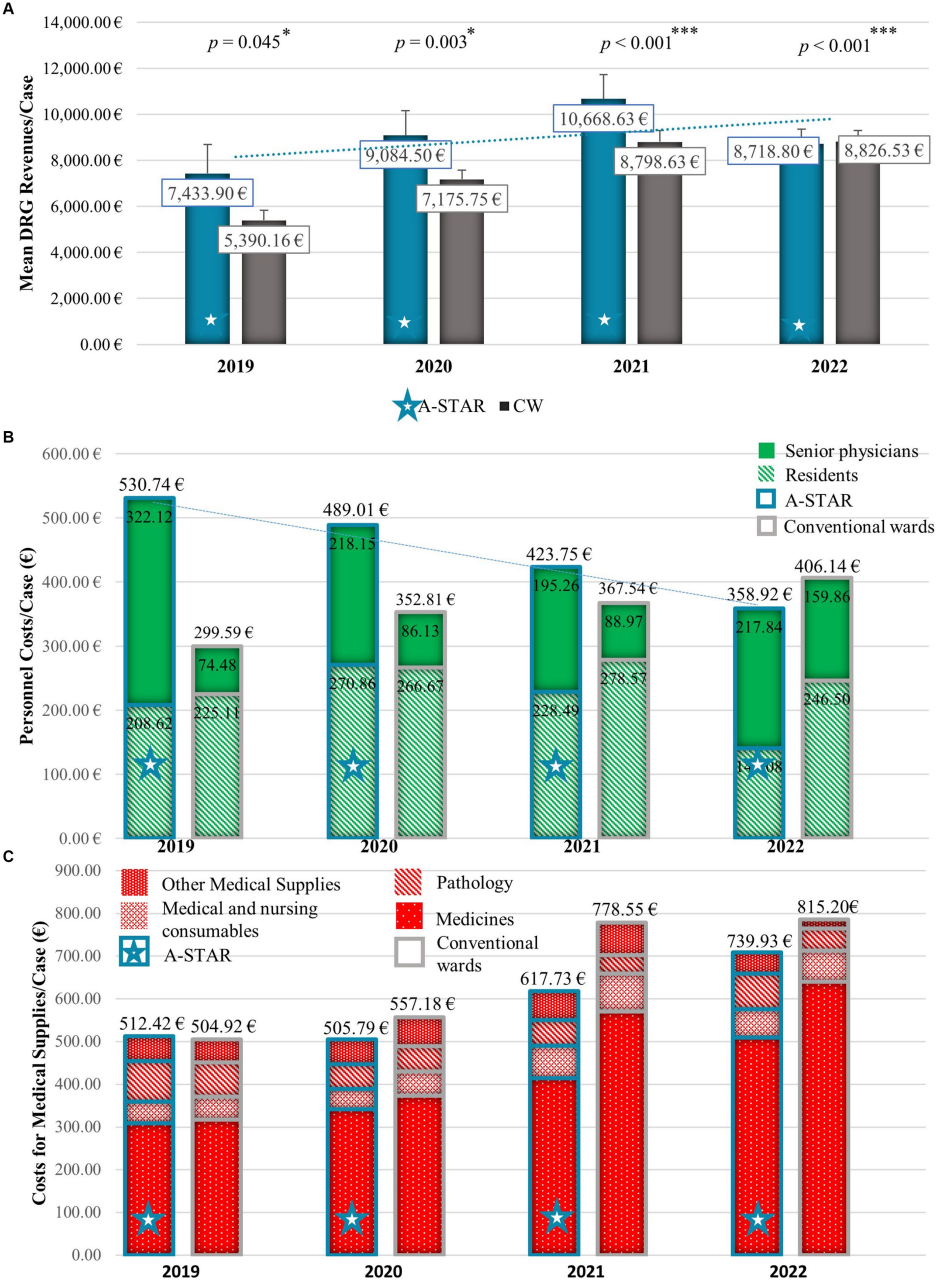
The interprofessional training ward A-STAR (blue star) performs superior over conventional wards concerning mean annual Diagnosis Related Groups (DRG) revenues per case and material costs per case and slightly higher personnel costs per case. **(A)** Mean annual DRG revenues/case with standard error of the mean of the A-STAR compared to conventional wards. On average, A-STAR (blue star) generated significantly higher revenues per case compared to conventional wards (grey). 2019: €7,433.90 (n = 78) vs. €5,390.16 (*n* = 506); *U* = 16955.500; *Z* = −2.003; *p* = 0.045; 2020: €9,084.50 (*n* = 463) vs. €7,175.75 (*n* = 1759); *U* = 370964.000; *Z* = −2.951; *p* = 0.003;2021: €10,668.63 (*n* = 462) vs. €8,798.63 (*n* = 1737); *U* = 347221.500; *Z* = −4.454; *p* < 0.001;2022: €8,718.80 (*n* = 480) vs. €8,826.53 (*n* = 1717); *U* = 365450.000; *Z* = −3.795; *p* < 0.001. *p < 0.05% (significant), **p < 0.01% (very significant), ***p < 0.001% (highly significant). **(B)** Mean annual personnel costs per case for senior physicians (uniformly) and residents (hatched) of the interprofessional training ward (A-STAR) compared to conventional wards. The higher personnel costs per case of the interprofessional training station A-STAR (blue star) between 2019 and 2021 in comparison to conventional units were mitigated by a gradual increase in the number of beds allocated to A-STAR. **(C)** Mean annual materials costs per case (medicines, medical and nursing consumables, pathology, other medical supplies) of the interprofessional training ward (A-STAR) compared to conventional wards. With inflation, the material costs of the units increased between 2019 and 2022. After initially having slightly higher material costs in the case of A-STAR compared to conventional units, these costs, particularly medical expenses, were lower from 2020 to 2022.

**Figure 3:**
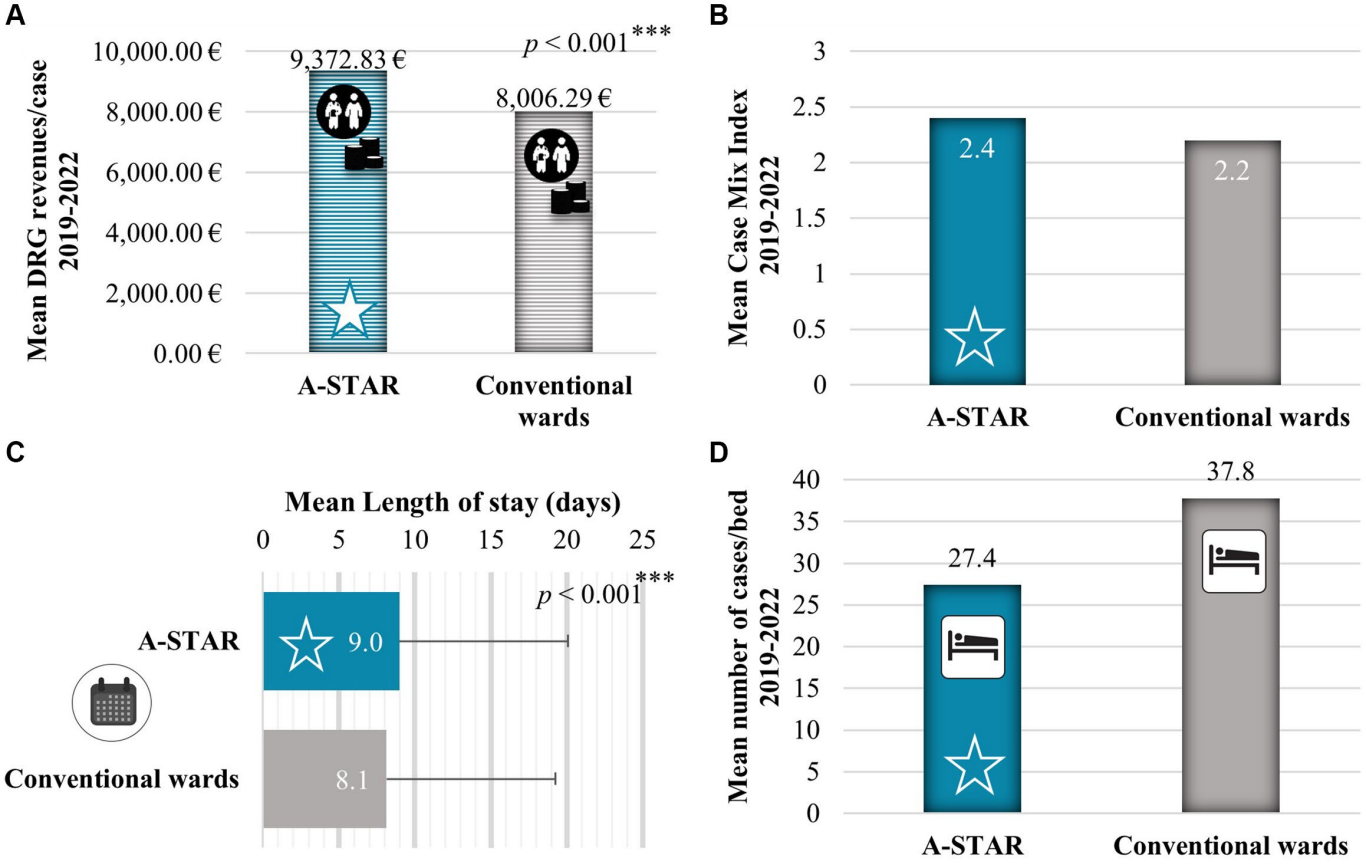
The interprofessional training ward A-STAR generated higher Diagnosis Related Groups (DRG) revenues through the treatment of patients with higher case complexity. **(A)** Between 2019 and 2022, A-STAR generated significantly higher revenues €9,372.83 per case on average (95% confidence interval [CI], €8,354.61 to €10,391.04.), as compared with the conventional wards with €8,006.29 per case (95% CI, €7,526.87 to €8,485.70); *U* = 3726527.000; *Z* = −7.205; *p* < 0.001. **(B)** In the period between 2019 and 2020, A-STAR (blue star) managed patients with greater case complexity and resource utilization, resulting in a higher Case-Mix Index (2.4 compared to 2.2) when compared to the conventional units (gray). **(C)** Mean length of stay in days of the interprofessional training ward A-STAR was very significantly longer compared to the conventional wards (9.0 vs. 8.1 days); *U* = 3925481.500; *Z* = −5.103; *p* < 0.001. **(D)** Between 2019 and 2022, A-STAR (blue star) handled fewer patient cases per bed compared to the conventional units (27.4 vs. 37.8). *p < 0.05% (significant), **p < 0.01% (very significant), ***p < 0.001% (highly significant).

On average, A-STAR generated significantly higher revenues €9,372.83 per case [95% confidence interval (CI), €8,354.61–€10,391.04], as compared with the conventional wards (*U* = 3726527.00; *Z* = −7.205; *p* < 0.001) with €8,006.29 per case (95% CI, €7,526.87–€8,485.70) during the years 2019 until 2022 (Figures 2A, 3A).

The personnel costs per case of the A-STAR were initially higher than the conventional wards but were reduced over time by increasing the number of beds in the A-STAR (2019: 8 beds; 2022: 12 beds) (Figure 2B). Despite the increase in salaries according to collective agreements, the personnel costs for the A-STAR senior physician per case could be reduced due to the increase in the number of beds on the A-STAR from 322.12€ (2019) to 217.84€ (2022) and the personnel costs for the A-STAR residents per case from 208.62€ (2019) to 141.08€ (2022).

The total material costs per case of the A-STAR were initially higher than the conventional wards (2019: €2278.00 vs. €2100.97) but already fell below the costs of conventional wards from 2020 (2020: €2196.47 vs. €2384.12; 2021: €727.66€ vs.914.64; 2021: €845.95 vs. €908.74). The abrupt drop in material costs across all stations between 2020 and 2021 is attributed to the gradual discontinuation of internal activity allocation. Internal activity allocation is a cost center accounting allocation method that allocates costs for internal activities to the department that incurred them, e.g., laboratory and radiological diagnostics. On annual average, the total material costs on the A-STAR per case were €1512.02 and on the conventional wards €1577.12. The team can mainly influence medical (Figure 2C) internal activity allocation (IAA) costs. The mean total medical costs (A-STAR: €739.93 vs. CW: €815.20) per case in particular for medicines (A-STAR: €393.61 vs. CW: €475.63) but also medical and nursing consumables (A-STAR: €60.30 vs. CW: €67.91) lay beyond the costs of the conventional wards (Figure 3B). A-STAR spent more than conventional wards on pathology (A-STAR: €73.69 vs. CW: €58.43) and consultation of physicians with other specializations (€17.43 vs. €13.12) per case. IAA showed no relevant differences per case between A-STAR and conventional wards (€803.27 vs. €801.97).

### 3.3 The interprofessional training ward A-STAR: generating higher DRG revenues for complex cases

The higher DRG revenues of A-STAR were generated through the treatment of more complex cases than the conventional wards (Figures 3A,B). In the German Diagnosis Related Group (DRG) system, the higher revenue is the more economically severe the illness of the patient case. The economic severity of illness is represented by the relative weight multiplied by the base rate to obtain the DRG revenue. The Institute for Hospital Remuneration (InEK) sets the prime rate. The case-mix index (CMI) is a direct indicator of case severity. It is calculated by dividing the additive total of all relative weights by the additive total of treatment cases. The average CMI is higher in A-STAR (2.4) than in conventional wards (2.1) (Figure 3B). The increased case complexity results in longer lengths of stay (Figure 3C) and subsequently lower case numbers in a year at A-STAR (Figure 3D). The mean length of stay was longer at A-STAR compared to conventional wards (9.0 ± 11.1 vs. 8.1 ± 11.6 days, *U* = 3925481.500; *Z* = −5.103; *p* < 0.001). Per bed, A-STAR (27.4 cases/year) treats fewer patients than conventional wards (37.8 cases/year).

### 3.4 The interprofessional training ward A-STAR: balancing slightly higher personnel costs with increased DRG revenues and efficient material expenses

**Figure 4:**
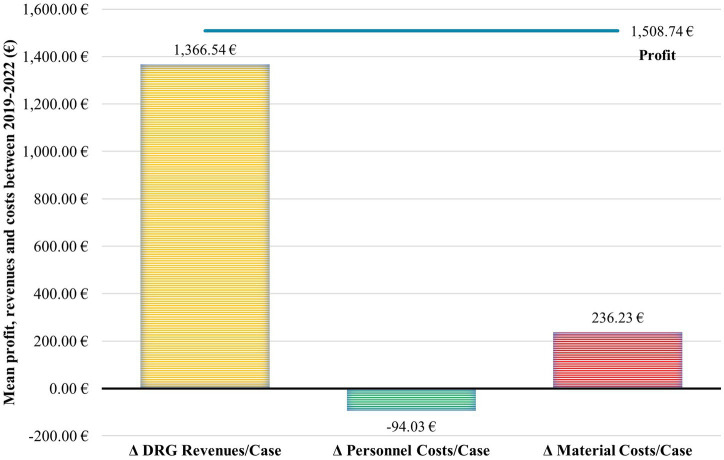
The slightly higher personnel costs of A-STAR are offset by the higher DRG revenues per case and the more resource-efficient material expenses. Between 2019 and 2022, A-STAR achieved on average €1,508.74 profit more per case compared to conventional units. This gain can be attributed to A-STAR having higher Diagnosis Related Group (DRG) revenues per case (€1366.54 difference to DRG of conventional wards) and lower material costs per case (€236.23 difference to material costs of conventional wards) and only slightly higher personnel costs per case (€94.03 difference to the personnel costs of conventional wards).

Between 2019 and 2022, A-STAR realized an average profit increase of €1,508.74 per case compared to traditional units (Figure 4). This boost in profit can be attributed to A-STAR’s higher Diagnosis Related Group (DRG) revenues per case (€1,366.54 higher than DRG revenues in conventional wards) and lower material costs per case (€236.23 less than material costs in conventional wards), along with only slightly higher personnel costs per case (€94.03 more than personnel costs in conventional wards).

### 3.5 The interprofessional training ward A-STAR: surpassing the bed occupancy of conventional wards for patients requiring isolation

**Figure 5:**
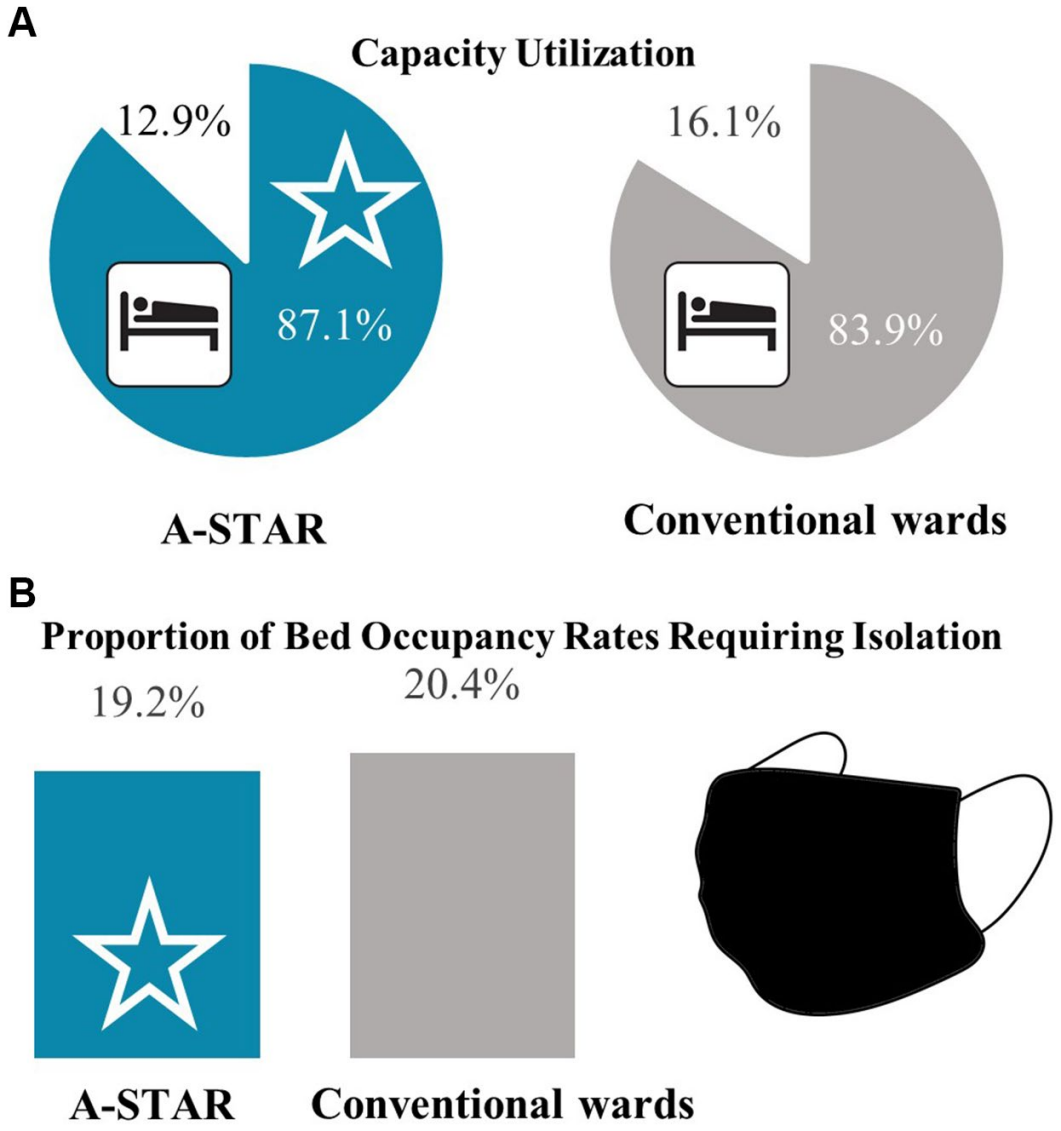
The bed occupancy rate of the interprofessional training ward A-STAR surpassed that of the conventional wards with a comparable proportion of patients requiring isolation. **(A)** Between 2019 and 2022, A-STAR (blue star) demonstrated a higher capacity utilization compared to the conventional units (87.1% vs. 83.9%). **(B)** The proportion of bed occupancy days requiring isolation was comparable between the interprofessional training ward A-STAR and conventional wards (CW); χ^2^(1) = 0.985; *p* = 0.321.

At 87.1%, the capacity utilization rate of A-STAR was higher than that of conventional wards (83.9%) (Figure 5A). The A-STAR consists of double-occupancy rooms, the conventional wards have, in addition to four single rooms, exclusively double-occupancy rooms as well. When patients cannot be cohort-isolated due to mandatory isolation of specific pathogens, it results in unoccupied beds. A-STAR showed a similar proportion of bed days with patients requiring isolation [19.2% vs. 20.4%; χ^2^(1) = 0.985; *p* = 0.321], who may lead to bed vacancies if they cannot be cohort-isolated in the double bedrooms (Figure 5B).

### 3.6 The interprofessional training ward A-STAR: demonstrating superiority in recruiting medical and nursing trainees for deployment

**Figure 6:**
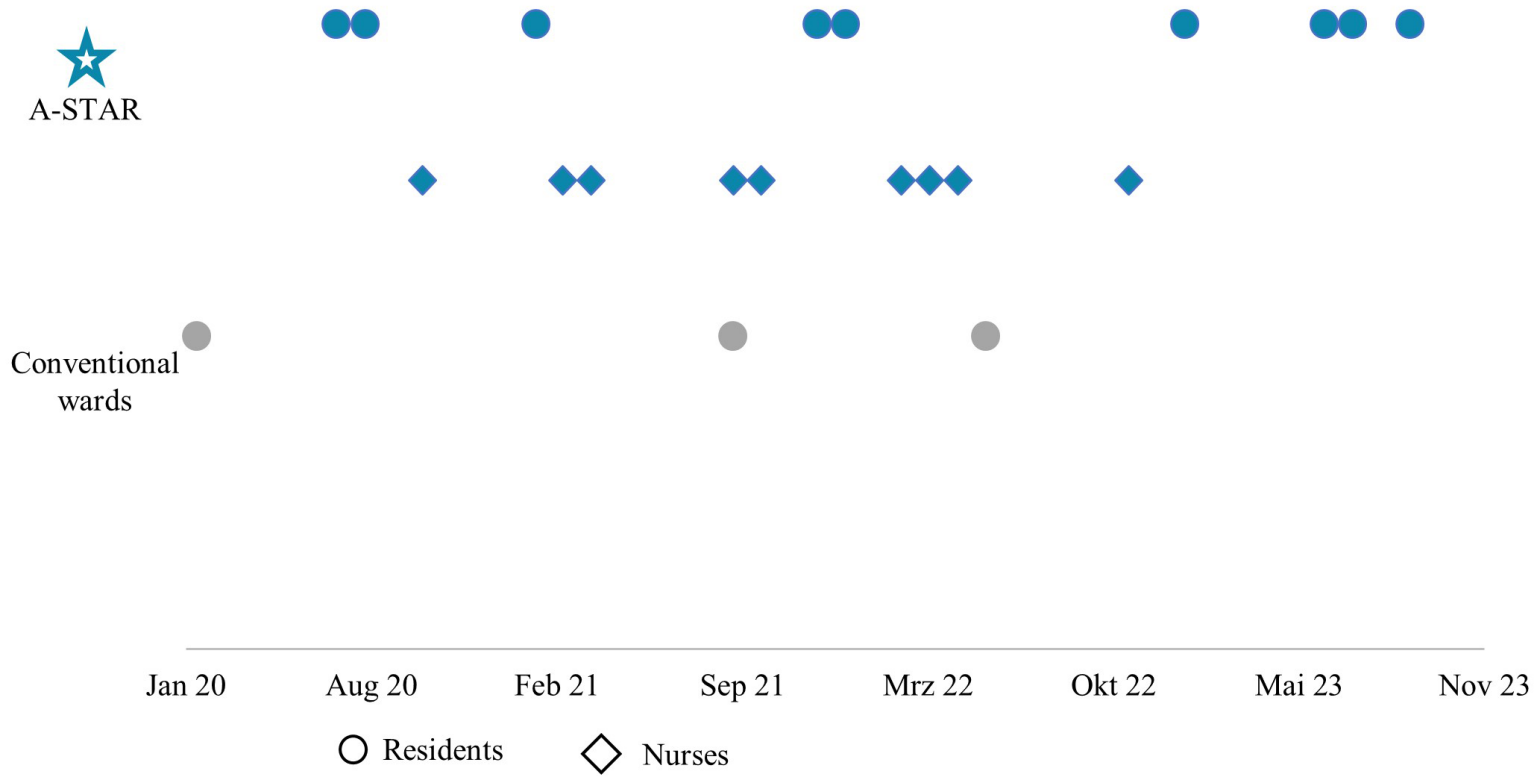
The interprofessional training ward A-STAR clearly excels in terms of recruiting medical (dot) and nursing (diamond) staff from the pool of trainees, who had been deployed on A-STAR and conventional wards. Between January 2020 and November 2023, a total of 9 doctors and 9 nurses who had been deployed at the A-STAR in advance started their first jobs in the Department for Internal I at the university hospital. In contrast, only 3 doctors from their trainee pool of the conventional wards were hired during this period.

The earliest possible hiring start date after deployment on the A-STAR was January 1, 2020. Since then, notably, more new residents (9 vs. 3) and nurses (9 vs. 0) were recruited from the pool of medical students and nursing trainees who had worked in the A-STAR than the pool of medical students and nursing trainees from the conventional wards (Figure 6).

## 4 Discussion

Interprofessional training wards offer an exclusive opportunity for healthcare professionals to enhance their skills, collaborate, and learn in a real clinical setting. The scarcity of cost-efficiency data regarding these training wards hinders their adoption. We embarked on an investigation to explore the hypothesis that an interprofessional training ward, integrated within a university’s internal medicine department, operates with comparable cost efficiency to conventional wards. The data presented in this comprehensive analysis shed light on the outstanding performance of the interprofessional training ward, A-STAR, in several key aspects of healthcare management. This discussion delves into the various findings, highlighting their implications and significance in the context of healthcare delivery and resource management.

One of the standout achievements of A-STAR is its superior financial performance per case. Over the years from 2019 to 2022, A-STAR consistently generated substantially higher annual DRG revenues per case, outperforming conventional wards by €1,366.54 on average. A-STAR was able to generate higher DRG revenues by treating cases with greater complexity. The higher case complexity of the patients treated at A-STAR justifies the longer length of stay, which also resulted in a lower number of cases treated. The data indicates that A-STAR had a higher case-mix index (CMI), reflecting the complexity of the cases they handled. As a result, patients at A-STAR required a longer length of stay on average, which is a reasonable outcome given the need for more extensive care. In retrospective cohort studies conducted by Hansen et al. (30) and Kuner et al. (31), surgical and orthopaedic training wards exhibited shorter postoperative stays than conventional wards but showed no significant difference in baseline characteristics and probably case severity between their interprofessional training wards and their conventional wards. While randomization was not formally executed, one might have anticipated that the medical team would assign less complex cases to learners within A-STAR. This was probably unfeasible due to high occupancy. The higher CMI suggests that the medical performance of the A-STAR team allowed for severe cases to be assigned to the ward. A previous study about the A-STAR revealed that patient outcomes in the A-STAR ward were comparable to those in conventional wards, with similar rates of discharges against medical advice, complication-driven readmissions, ICU transfers, and mortality (32). Additionally, the high levels of patient satisfaction, particularly regarding team competence, ward atmosphere, and responsiveness to concerns, highlight the positive impact of interprofessional collaboration and education on patient care. These findings suggest that the structured interprofessional environment of A-STAR contributes significantly to its medical performance.

A-STAR also managed to reduce material costs per case over time. Certainly, particularly noteworthy is the significant reduction in medication expenses at the A-STAR. This could potentially be attributed to the regular oversight provided by pharmacy colleagues. Preliminary research indicates that collaborative efforts among pharmacists, nurses, and physicians can effectively curtail antibiotic expenses (33). The additional cost of A-STAR for pathology and consultation with physicians in other specialties aligns with the team’s level of training. Collectively, these costs form a minor fraction of the overall material expenses and are justifiable considering the valuable learning outcomes they yield. Consistently, Hansen et al. found lower overall costs for treatment with a hip replacement in their interprofessional training ward than in their conventional wards (30) but did not explicitly break these down into material costs.

Despite the higher case complexity, A-STAR was not compromised by an increase in isolations and, in fact, demonstrated a higher bed occupancy rate compared to conventional wards. This observation is significant because treating more complex cases often involves a higher likelihood of isolation requirements, which can potentially lead to unoccupied beds due to infection control measures. However, the data suggests that A-STAR effectively managed patient isolations and maintained a higher bed occupancy rate, which is indicative of efficient resource utilization.

This increase in revenue per case over conventional wards contributed to a substantial boost in profit despite higher personnel costs per average. The A-STAR program incurred elevated personnel costs per case, primarily due to the enhanced oversight offered by the senior physician dedicated to A-STAR, in contrast to their counterparts in the conventional wards. Similarly, Hansen et al. found increased staffing expenses in their orthopedic interprofessional training facility compared to conventional wards (30). It is imperative to underscore that patient safety remains paramount in training. As such, any compromise on the presence of senior physicians in the pursuit of cost reduction is unequivocally unacceptable. The assurance of patient well-being stands as a non-negotiable principle in this context. The personnel costs decreased as the number of beds in A-STAR increased. This suggests that scaling up the ward can be a viable strategy to optimize personnel costs.

An additional positive outcome observed during the study was the successful recruitment of medical staff, attributed to the engagement of trainees and students, although this was not the primary objective of the study. A-STAR demonstrated superior recruitment of medical and nursing trainees, a critical component of medical education and workforce development. According to the World Health Organization’s State of the World’s Nursing 2020 report, a significant global shortage of approximately 6 million nurses is by anticipated by 2030 (34). This shortage has already led to unoccupied hospital beds, and there is a growing scarcity of doctors. The trend of physicians choosing part-time schedules due to increasingly compressed work hours exacerbates this challenge, necessitating a larger workforce. In light of these challenges, the organization’s remarkable success in personnel acquisition is encouraging. This achievement is likely influenced by a simultaneous sense of profound satisfaction stemming from both educational pursuits and professional endeavors. To gain a more comprehensive understanding of this phenomenon, it is essential that this relationship is subjected to further investigation in subsequent research endeavors.

The significant financial advantage of A-STAR and its success in recruiting healthcare workers is not only noteworthy but also plays a vital role in the sustainability of interprofessional training wards. The ability to achieve higher revenues while providing quality care reflects positively on the effectiveness of interprofessional training wards despite their educational mission. The study period coincided with the impact of the Covid-19 pandemic, officially declared on March 1, 2020. The pandemic-related restrictions persisted in Germany until April 7, 2023. Medical education largely shifted to digital platforms (24, 35–39), with negative impacts on students’ psychological well-being (40–42). However, training of medical students and nursing trainees in the A-STAR remained uninterrupted during the pandemic without compromising revenue. In fact, the department’s Diagnosis-Related Group (DRG) revenue increased during this challenging period. This underscores the resilience and economic viability of interprofessional training wards, even when facing exceptional circumstances.

Spanning more than 3 years and scrutinizing 7,234 patient cases, this study presents a comprehensive perspective on the cost-effectiveness of a training ward vis-à-vis conventional wards. A notable advantage of this research lies in including a control group comprising conventional wards. The study ensures real-world data analysis from a diverse internal medicine patient cohort, steering clear of artificial constraints associated with a single-case focus.

Nonetheless, certain limitations warrant consideration. Notably, the study’s scope could be more expansive in its ability to delve into qualitative outcome parameters of internal medicine interventions. Additionally, it is essential to acknowledge that material costs were not individually tracked per case but instead were derived in total, not per case basis, within the account of one of the conventional wards and allocated based on the number of beds. Another limitation of this study is that it did not account for the costs associated with the organization and coordination of the interprofessional training ward. However, as it stands, this remains the sole instance of a comprehensive breakdown of revenue and expenditures for an interprofessional training station when contrasted with conventional wards.

Our findings suggest that in addition to their recognized advantages, interprofessional training wards offer cost-effectiveness. This discovery may serve as a compelling rationale for the wider implementation of such educational facilities. Establishing interprofessional training wards on a wide scale is advisable as breeding grounds for upcoming professionals. Future research should examine quantitative outcome parameters of heterogeneous patient cohorts from interprofessional training wards and the achievement of learning objectives by the trainees.

## Data availability statement

The raw data supporting the conclusions of this article will be made available by the authors, without undue reservation.

## Ethics statement

The studies involving humans were approved by the Universität Regensburg Ethikkommission 93040 Regensburg. The studies were conducted in accordance with the local legislation and institutional requirements. Written informed consent for participation was not required from the participants or the participants' legal guardians/next of kin in accordance with the national legislation and institutional requirements.

## Author contributions

SS-H: Conceptualization, Data curation, Formal analysis, Investigation, Methodology, Project administration, Supervision, Validation, Visualization, Writing – original draft, Writing – review & editing. EA: Data curation, Investigation, Writing – review & editing. MM: Data curation, Investigation, Writing – review & editing. SA-F: Writing – review & editing. AMa: Investigation, Writing – review & editing. KR: Writing – review & editing. SR: Writing – review & editing. BM: Writing – review & editing. AMe: Writing – review & editing. CK: Writing – review & editing. SS: Writing – review & editing. MM: Conceptualization, Funding acquisition, Project administration, Resources, Supervision, Validation, Writing – review & editing.
